# Risk Prevention and Control Points Through Quantitative Evaluation of *Campylobacter* in a Large Broiler Slaughterhouse

**DOI:** 10.3389/fvets.2020.00172

**Published:** 2020-04-22

**Authors:** Ge Zhao, Xiumei Huang, Jianmei Zhao, Na Liu, Yuehua Li, Lin Wang, Yubin Gao, Juan Wang, Zhina Qu, Junhui Liu, Junwei Wang

**Affiliations:** Department of Pathogenic Microorganisms, China Animal Health and Epidemiology Center, Qingdao, China

**Keywords:** quantitative risk evaluation, broiler, slaughtering process, *Campylobacter*, critical control point

## Abstract

Chickens contaminated with *Campylobacter* are a major risk factor for human *Campylobacter* disease. As a result of the slaughter process, infections should be strictly controlled due to complete exposure of the chickens and the cross-contamination of pathogens. Using @RISK software, quantitative evaluation models of *Campylobacter* contamination during slaughtering in a large broiler slaughterhouse were constructed. Broiler scalding was set as the starting point of evaluation and four major processes including defeathering, eviscerating, pre-cool rinsing, and splitting-transmission were included. Through the simulation of the constructed model, 90% probability of *Campylobacter* in 100 g chickens after slaughtering were distributed between 0.3 and 50.2 MPN, which was consistent with simulated actual monitoring data 0–16.6 MPN, indicating that the model shows high credibility. In addition, growth curves of *Campylobacter* during whole slaughtering showed that contamination significantly increased after defeathering, and increased again after pre-cool rinsing. Using correlation coefficients to analyze the sensitivity of each parameter in the model, it was determined that the concentration of *Campylobacter* in the pre-cool pond water (correlation coefficient: 0.95) was the most critical risk point of sanitary control in this slaughterhouse. In conclusion, this study is the first to incorporate environmental factors during broiler slaughtering into the risk evaluation of *Campylobacter* contamination, which provides guidance for the sanitary control and risk management of *Campylobacter* contamination during broiler slaughtering.

## Introduction

The colonization rates of *Campylobacter* in the intestine, as a symbiotic bacteria in the intestinal tract of broilers, can reach 90%, but broilers show minimal clinical symptoms even when heavily infected (1). Broilers are easily contaminated by *Campylobacter* viscera when carrying recessive *Campylobacter* when entering the slaughtering stages due to complete exposure to the environment during slaughtering, leading to a wider cross-contamination and *Campylobacter* epidemics in chicken. As a result, 55.4% of retail chickens are contaminated by *Campylobacter* according to the latest surveys by the Food Standards Agency (FSA) in 2019 (2). A survey by the Federal Consumer Protection and Food Safety Agency (BVL) in 2017 found that 51.8% of retail chickens were contaminated by *Campylobacter*, and that 78.8% of slaughterhouse chickens are contaminated (3). Meanwhile, the contamination rates of *Campylobacter* in the chicken produced from large slaughterhouses in Eastern China was as high as 36.7% in 2018 (Monitoring data from our lab). It has been reported that 20–30% of *Campylobacter* cases are related to consumption of chickens contaminated with *Campylobacter* (4). Controlling *Campylobacter* during broiler slaughter therefore represents an effective means to prevent the risk of *Campylobacter* disease in the population.

Quantitative risk evaluation technology, as the optimal model of microbial risk evaluation, can provide important data references for the formulation of risk management policies (5). Combining microbial quantitative risk evaluations with critical control points can effectively reduce the cross-contamination of pathogenic bacteria. In this study, critical risk control points of *Campylobacter* contamination in a large-scale broiler slaughterhouse (the mainstream mode of broiler slaughtering in China) were analyzed using quantitative evaluation technology to provide guidance for the more targeted control of *Campylobacter* contamination during the slaughtering process.

## Materials and Methods

### *Campylobacter* Contamination Data in a Large Broiler Slaughterhouse

The parameters of broiler slaughter and processing were obtained through on-site investigations or expert consultations during the sampling process. Meanwhile, data on the contamination of *Campylobacter* in all stages of slaughter were obtained from monitoring data in our laboratory in the “Quality and Safety Risk Assessment Project of Animal and Poultry Products” of the Ministry of Agriculture in 2018. A total of 270 samples of *Campylobacter* were collected from the four links of scalding, rinsing, pre-cool rinsing, and segmental transmission in a large broiler slaughterhouse in the Shandong Province. As a result, *Campylobacter* was isolated, cultured and identified. Quantitative and qualitative results of *Campylobacter* contamination in chickens, viscera and the environment were obtained.

### Quantitative Risk Assessment

In this study, risk assessment software @RISK 7 were used to fit the random distribution of data through its distribution fitting functiona. Meanwhile, variables and parameters involved in risk evaluations were expressed by specific values, formulas, or distributions. Data was input in Excel worksheet and models were built using @RISK 7. Monte Carlo simulation was performed using the Latin hypercube sampling method. Random computer extracts and probability distributions were obtained from each time point in simulations of 10,000 iterations.

### Construction of Quantitative Risk Assessment Model

A slaughter batch of broiler chickens, with the number of 50,000–150,000 usually, were assessed. Broiler chickens are directly exposed to air and environmental utensils after scalding, so post-scalding was taken as the starting point of the evaluation process. Defeathering, eviscerating, pre-cool rinsing, and splitting-transmission were the four processes that were successively passed downstream.

### Defeathering

The contamination of *Campylobacter* after hot washing was the initial value used for the evaluation model. After hot washing, the majority of bacteria on the chicken surface have been inactivated, and quantitative data cannot be detected directly. The contamination of *Campylobacter* in samples is converted from qualitative to quantitative data by the formula M = –(2.303/V) × lg (Nneg/Ntotal) (6). Amongst the values, V refers to the volume diluted by 100 cm^2^ cotton swab sample, Nneg refers to the number of negative samples detected, and Ntotal refers to the total number of samples. The defeathering process of the eviscerating machine can cross-contaminate the remaining *Campylobacter* to other chickens, and as a consequence, the contamination status of the eviscerating machine can be assessed as a risk contribution factor in the model. The contamination density of the eviscerating machine calculated through the cumulative probability distribution M = Cumulative (Min, Max, {a1, a2, a3…}, {p1, p2, p3…}) Fitting, in which Min is the minimum, Max is the maximum, a is the levels of *Campylobacter* (1 CFU = 1MPN), and p is the cumulative probability corresponding to the amounts of *Campylobacter*. Furthermore, it was assumed that half of the *Campylobacter* in the chicken body contacting the surface can be transferred to the chicken body in each batch of the defeathering machine, with total contamination amounts (N1) on the carcass of broiler chickens after scalding being the sum of *Campylobacter* remaining after scalding and newly contaminated after defeathering. All parameters are shown in Table 1.

**Table 1:** Parameter settings of *Campylobacter* quantitative risk evaluation models for broiler slaughter.

**Assessment module**	**Description**	**Symbol**	**Unit**	**Distribution or formula**	**Source**
Scalding-defeathering	Number of chickens in one batch	Np		Uniform (50,000, 150,000)	Investigation
	Pre-slaughter surface area of single chicken	M1	100cm^2^	Pert (10, 12, 14)	Measure
	*Campylobacter* carried after scalding	Pp		Discrete ({0, 1}, {0.967, 0.033})	Monitoring
	Density of *Campylobacter* carrying after scalding	Mn	MPN/100cm^2^	Poisson [10^(−2.303/5)*lg (96.7/100)^]	
	Total pollution after scalding	N1a	MPN	IF (Pp = 0, Mn*m1*Np, Mn*m1*Np)	
	*Campylobacter* carrier rates of feather removal machine	Pt		Discrete ({0, 1}, {0.484, 0.516})	Monitoring
	*Campylobacter* density of the feather removal machine	Mtn	MPN/100cm^2^	Cumulative (4, 72, {4, 23, 37}, {0.222, 0.555, 0.778})	
	Predicted contact area of the eviscerating machine	Mt	100cm^2^	Pert (1,600, 3,200, 4,800)	Measure
	Number of chickens in one depilation batch	Npt		Pert (60, 120, 180)	Investigation
	*Campylobacter* quantity of chicken carcass cross-contaminated by the feather remover	N1b	MPN	0.5*IF (Pt = 0, 0, Mtn*mt*Np/Npt)	
	Total pollution after hair removal	N1	MPN	N1 = N1a+N1b	
Eviscerating	*Campylobacter* carrier rates on the intestinal surface	Pg		Discrete ({0, 1}, {0.536, 0.464})	Monitoring
	Intestinal surface *Campylobacter* load	Mg	MPN/ chicken	Cumulative (3, 33, {3, 5, 15}, {0.273, 0.636, 0.818})	Monitoring
	Transfer of intestinal surface *Campylobacter* to the chickens	N2a	MPN	0.2*IF (Pg = 0, 0, Mg*Np)	
	Hand-borne *Campylobacter* rates of workers	Ph1		Discrete ({0, 1}, {0.367, 0.633})	Monitoring
	Hand-borne *Campylobacter* levels of the workers	Mh	MPN/hand	Cumulative (5, 66, {5, 14, 28}, {0.385, 0.538, 0.846})	Monitoring
	Number of workers	Nh1		Pert (20, 60, 100)	Investigation
	Transfer of *Campylobacter* from the chicken to the workers' hands	N2b	MPN	0.5*IF (Ph1 = 0, 0, Mh*Nh1*Np/Nh1)	
	*Campylobacter* carrying rates of the tools	Pj		Discrete ({0, 1}, {0.5, 0.5})	Monitoring
	*Campylobacter* carrying capacity of the tools	Mj	MPN/ handle	Cumulative (6, 33, {6, 20, 33}, {0.167, 0.5, 1.0})	Monitoring
	Number of tools	Nj		Pert (20, 120, 200)	Investigation
	Transfer of *Campylobacter* from tools to chicken	N2c	MPN	0.5*IF (Pj = 0, 0, Mj*Nj*Np/Nj)	
	Total *Campylobacter* contamination after eviscerating	N2	MPN	N2 = N1 + N2a + N2b + N2c	
Pre-cool rinsing	Pre-cool water volume for one chicken	V	mL	Pert (1,000, 1,200, 1,500)	Investigation
	*Campylobacter*-carrying rates in water in the pre-cool pond	Pc		Discrete ({0, 1}, {0.53, 0.47})	Monitoring
	Concentration of *Campylobacter* in the water of the pre-cool pond	Mnc	MPN/mL	Poisson [10^(−2.303/10)*lg (53/100)^]	
	Total *Campylobacter* contamination in the pre-cool pond	N3a	MPN	IF (Pc = 0, Mnc*V*Np, Mnc*V*Np)	
	Total *Campylobacter* contamination after pre-cool	N3	MPN	0.2* (N2 + N3a)	
Splitting- transmission	Total Cutting Tool Quantity	Nk		Pert (100, 200, 300)	Investigation
	*Campylobacter* carrying rates of the cutting tools	Pk		Discrete ({0, 1}, {0.625, 0.375})	Monitor
	*Campylobacter* carrying capacity of the cutting tools	Mk	MPN/ handle	Cumulative (3, 17, {3, 5, 10}, {0.273, 0.636, 0.818})	Monitoring
	Cutting tool *Campylobacter* transfer to chickens	N4a	MPN	0.5*IF (Pk = 0, 0, Mk*Nk*Np/Nk)	
	Number of workers	Nh2		Pert (200, 400, 1,000)	Investigation
	Hand-borne *Campylobacter* rates of workers	Ph2		Discrete ({0, 1}, {0.645, 0.355})	Monitoring
	Hand-borne *Campylobacter* levels of the workers	Mh2	MPN/ hand	Cumulative (3, 126, {3, 16, 60}, {0.6, 0.8, 0.9})	Monitoring
	Transfer of *Campylobacter* from workers' hands to chicken	N4b	MPN	0.5*IF (Ph2 = 0, 0, Mh2*Nh2*Np/Nh2)	
	Conveyor belt contact area (1/10 Contact Surface)	S	100cm^2^	Uniform (50*120/10, 50*240/10)	Investigation
	Conveyor belt *Campylobacter* rates	Ps		Discrete ({0, 1}, {0.882, 0.118})	Monitoring
	Density of *Campylobacter* in the conveyor belt	Ms	MPN/100cm^2^	Cumulative (1, 27, {1, 12, 27}, {0.5, 0.75, 1.0})	Monitoring
	Number of chickens transported in one batch	Ns		Uniform (240, 480)	Investigation
	Transfer of *Campylobacter* from the conveyor belt contact to the chicken	N4c	MPN	0.5*IF (Ps = 0, 0, Ms*S*Np/Ns)	
	Total *Campylobacter* contamination after segmented transmission	N4	MPN	N4 = N3 + N4a + N4b + N4c	
	Post slaughter weights of single chicken	M	100g	Pert (14, 16, 18)	Investigation
	100 g chicken contamination after split transmission	N5	MPN/100g	N5 = (N4/Np)/M	

### Eviscerating

The intestinal tract of the broilers can carry *Campylobacter* recessively, and the viscera is easily punctured by the cutter during the eviscerating process. Residual feces in the cloacal orifice can easily contaminate the carcass of other broilers. Therefore, the viscera of chickens, workers' hands and eviscerating tools were considered as risk contributors. The increase of *Campylobacter* contamination is determined by the bacterial carrying rates, bacterial carrying capacity, the transmissibility of the visceral surface, workers' hands surfaces, and eviscerating tools. Meanwhile, the three risk contributing risk factors can detect the positive quantitative data of *Campylobacter*, and as a consequence, the bacterial carrying capacity is fitted by the cumulative probability distribution. The carrying rates are fitted by discrete probability distributions *p* = Discrete ({0, 1}, {Pneg, Ppos}), of which 0 represents negative, 1 represents positive, Pneg is negative rate, and Ppos is positive rate.

### Pre-cool Rinsing

Pre-cooling after slaughter can reduce the total contamination of *Campylobacter*, but increases the chance of cross contamination of *Campylobacter* when all broiler carcasses are collected in the pre-cool pond. Meanwhile, according to investigations in the broiler slaughterhouse, the volume of pre-cool water corresponding to a broiler is 1–1.5 L. Although the isolation rates of *Campylobacter* in pre-cool water are high, effective positive quantitative data for direct counting are not obtained. Therefore, the concentration of *Campylobacter* in the pre-cool pond water was also converted into quantitative data by the formula. The transfer rate of *Campylobacter* contaminated in the pre-cool pond to the carcass of broilers was calculated by 1/5 (7).

### Splitting-Transmission

Splitting-transmission is one of the most complex steps during broiler carcass exposure. Cutting tools, workers' hands and conveyor belts are all included in the evaluation. The number of carcass contact surfaces between the three risk contributing factors and broilers were obtained through on-site investigation. In addition to the positive isolation rates of *Campylobacter* fitted by discrete probability distributions, the positive quantitative data were fitted by the cumulative probability distribution. The transmissibility of *Campylobacter* to chickens through the conveyor belt were calculated by 1/2 (7). The total contamination of *Campylobacter* in one slaughter batch of chickens after splitting-transmission was expressed as N4, and those of *Campylobacter* in 100 g of chickens was expressed as N5.

### Probability Distribution of Actual Contamination of *Campylobacter* in Chicken Meat

Cotton swab samples directly smeared from 100 cm^2^ surface of post-slaughtered chickens were collected for qualitative and quantitative detection. Quantitative data are also fitted using the cumulative probability distribution (Table 2). The relationship between the surface area and the weight of the single chickens were converted by the formula m = 2^*^ (0.67^*^M^*^100+536) (8), in which M was the weight of single chickens.

**Table 2:** Fitting parameters of the probabilistic distribution of *Campylobacter* contamination in chickens.

**Description**	**Symbol**	**Unit**	**Distribution or formula**	**Source**
Surface area of single chicken after segmentation	M2	100cm^2^	2* (0.67*M*100+536)/100	((8))
Contamination rate of post-slaughter chickens	Pa		Discrete ({0, 1}, {0.846, 0.154})	Monitoring
Contamination density of *Campylobacter* in post- slaughter chicken	Ma	MPN/100cm^2^	Cumulative (1, 25, {1, 5, 15}, {0.133, 0.6, 0.933})	Monitoring
Contamination of 100 g chickens after slaughter	N6	MPN/100g	IF (Pa = 0, 0, Ma*m^2^/M)	

## Results and Analysis

### Quantitative Evaluation Models to Simulate the Contamination Probability Distribution of *Campylobacter* During Broiler Chickens Slaughtering

**Figure 1:**
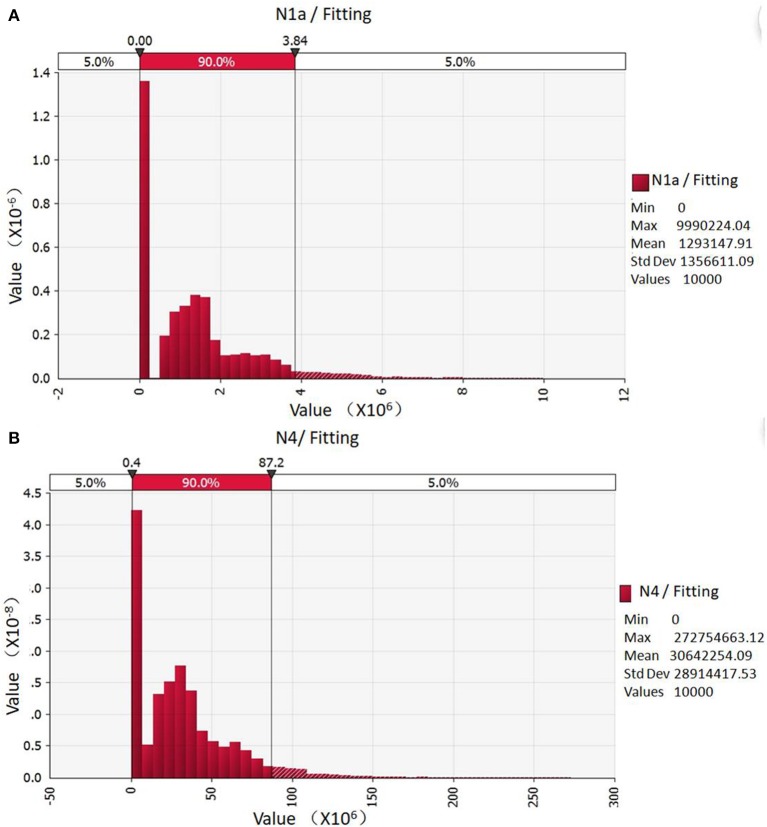
Probabilistic distribution of *Campylobacter* contamination in one patch of slaughtered broilers. **(A)** Initial total contamination of *Campylobacter*; **(B)** The total contamination of *Campylobacter* after segmentation simulated by evaluation Model.

The fitting data are shown in Figure 1A. The total contamination of *Campylobacter* in one batch broiler chickens after scalding was taken as the initial value, although the 90% probability was 0 MPN, the maximum value reached to 3.84 × 10^6^ MPN. Through quantitative risk assessment models of *Campylobacter* contamination in slaughter chickens, after defeathering, eviscerating, pre-cool rinsing, and splitting-transmission, 90% of the total *Campylobacter* contamination in broiler chickens was distributed between 0.4 × 10^6^ and 87.2 × 10^8^ MPN (Figure 1B), with an average of 3.06 × 10^7^ MPN. As a result, the slaughter process significantly increased the risk of *Campylobacter* contamination in chickens.

### Practical Verification of the Output

**Figure 2:**
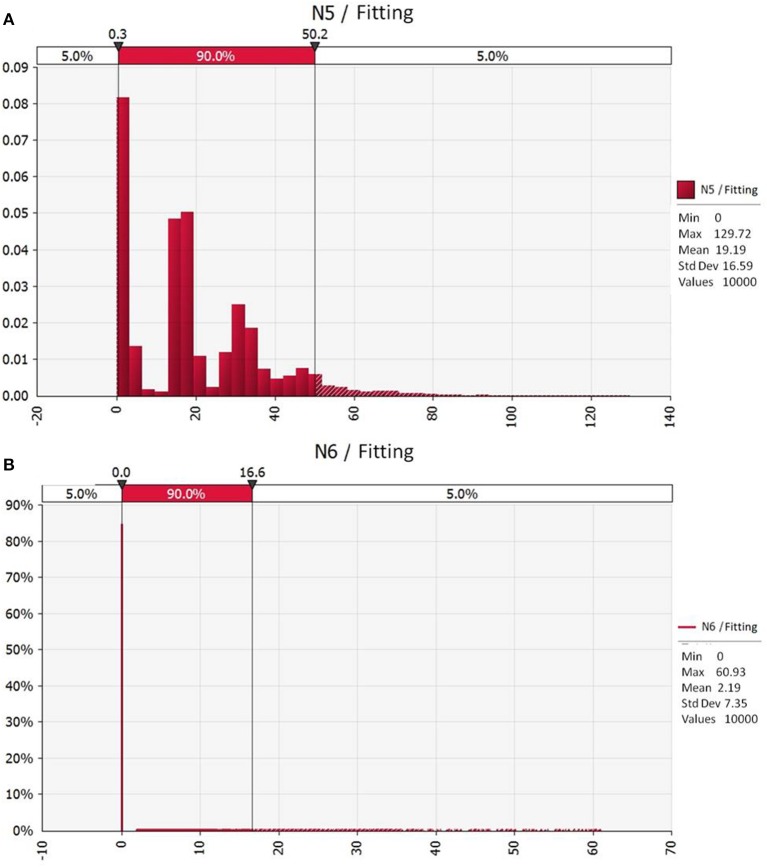
Distribution of *Campylobacter* contamination in 100 g of chicken after slaughtering. **(A)** Output results of the model simulation; **(B)** Fitting results of the actual monitoring data.

Using the simulation of the quantitative evaluation model of *Campylobacter* during the slaughtering of broiler, up to 90% of the contamination of *Campylobacter* in 100 g of chicken was found to distribute between 0.3 and 50.2 MPN, with an average of 19.19 MPN (Figure 2A). In addition, after fitting the actual monitoring data of the chickens after slaughtering, it was found that the 90% probability distribution of *Campylobacter* contamination in 100 g of chicken was 0–16.6 MPN (Figure 2B). These data were consistent with the contamination levels simulated by the model, and its average value of 2.19 MPN, was also between 0.3 and 50.2 MPN, which indicating that the quantitative evaluation model showed good reliability.

### Changes of *Campylobacter* in Chickens During Slaughtering

**Figure 3:**
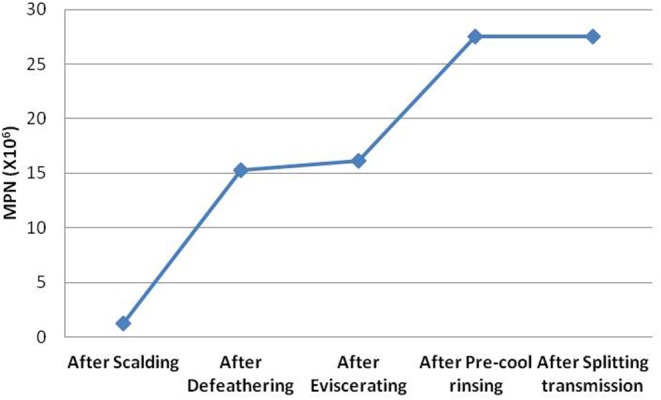
Changes of *Campylobacter* in broiler chickens during slaughtering. The average MPN of *Campylobacter* in the broiler chickens after defeathering, eviscerating, pre-cool rinsing and splitting-transmission was simulated, and the growth curves of *Campylobacter* contamination during slaughtering were constructed.

The total amounts of *Campylobacter* contamination in the broilers during defeathering, eviscerating, pre-cool rinsing, and splitting-transmission were further simulated through the quantitative evaluation model. According to the average MPN values obtained, the growth curve of *Campylobacter* carried by the broilers during slaughtering was constructed (Figure 3). Meanwhile, it was found that the contamination of *Campylobacter* significantly increased after defeathering, from 1.2 × 10^6^ to 15.3 × 10^6^ MPN, and then miner increased after eviscerating. The contamination of *Campylobacter* significantly increased after pre-cool rinsing again from 16.2 × 10^6^ to 27.6 × 10^6^ MPN, but then unchanged on the whole after splitting-transmission. It seems that the defeathering and pre-cool rinsing of broiler chickens in the slaughter house were the main risk contributors of *Campylobacter*.

### Analysis of Critical Risk Control Points in Broiler Slaughtering Process

**Figure 4:**
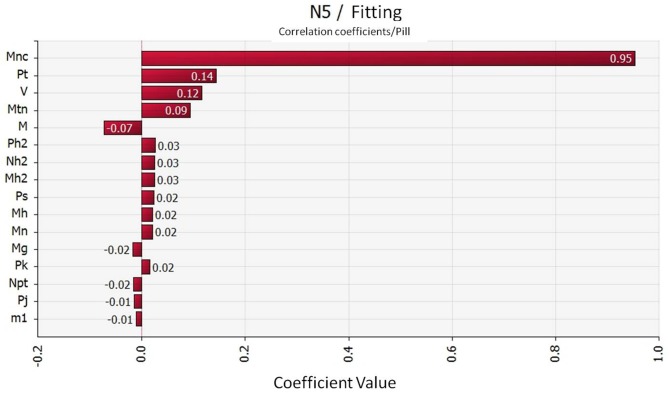
Coorelation coefficient of the *Campylobacter* hazard factors during slaughtering. Mnc: *Campylobacter* carrying concentration in the water of the pre-cool pond; Pt: *Campylobacter* carrying rate of defeathering machine; V: Volume of the pre-cool pond water; Mtn: *Campylobacte*r carrying capacity of defeathering machine.

The correlation between the contamination of *Campylobacter* in segmented chickens and the risk factors in the slaughtering process were discussed through sensitivity analysis of the parameters in the quantitative evaluation model. The risk contribution of each factor to the contamination of *Campylobacter* in the terminal chicken products were then determined. The results in Figure 4 show that the concentration carried in the water of pre-cool pond was the most critical risk point for *Campylobacter* contamination in the terminal chicken products (correlation coefficient value: 0.95). In addition, the carrier rate of *Campylobacter* on the defeathering machine was also contributed some risk to *Campylobacter* contamination in the terminal chickens (correlation coefficient value: 0.14).

### Uncertainty Analysis of the Quantitative Evaluation Model

The quantitative evaluation model of *Campylobacter* contamination during the slaughtering links of broilers constructed in the study had several uncertainties. An example is the uncertainty of the process and the model. This study assumed that *Campylobacter* does not proliferate during the slaughter process, despite its known ability to proliferate at the temperature before pre-cool rinsing. A further limitation is the uncertainty of quantitative data. Some parameters that failed to detect quantitative data were directly converted from the qualitative data, but remained unable to replace real quantitative data obtained from a large number of actual samples. The final uncertainty is from the empirical speculations. The majority of data originated from actual investigations and monitoring, but few were obtained through relevant experience speculation, such as the transfer rates of bacteria from tools and workers to the chickens. Here we assumed that half of the bacteria can be transmitted by contact, without the consideration of other factors.

## Discussion

Microbial quantitative evaluations hold high application values for the controlled measurement of risk probabilities and severities. Such predictions can prevent the exposure risk and hazard of pathogenic bacteria in food. The Monte-Carlo method simulates experimental results with a probability model, which better reflects the real risk situation (9). In this study, the quantitative evaluation method was used to study the contamination status of *Campylobacter* in each link of a large-scale broiler slaughterhouse, and the critical risk control points of the whole slaughtering process were identified to guide the supervision and control of microorganisms in the slaughterhouse more effectively.

China is a big country in chicken production and consumption. Current international data suggest that *Campylobacter* infections have become more serious (2, 3, 10) and represent a major challenge to food safety (11). Western developed countries have performed a systematic risk assessment on *Campylobacter* in chicken (12–14) and highlighted that the contamination rates of *Campylobacter* are proportional to the population incidence. Studies in China assessing the risk of *Campylobacter* from farms to dining tables in chicken have estimated the incidence of *Campylobacter* disease in the current contaminated population. In this study, 90% of *Campylobacter* contamination in chicken was estimated at between 0.4 × 10^6^ and 87.2 × 10^8^ MPN per 100 g, which was consistent with the actual monitoring data. Although this initial contamination is not sufficiently high to cause disease, *Campylobacter* is a living organism, if it exponentially proliferate under the hotter temperature in summer, it only takes a few hours to reach the pathogenic dose of 400–500 CFU (15).

Previous study (16) highlighted *Campylobacter* contamination in eviscerating link was the critical risk control point during the slaughtering process, however, the risk of the slaughtering environment to carcass cross-contamination was not analyzed. Chickens are completely exposed to the environment during slaughtering and can be directly cross-contaminated by pathogenic bacteria in the environment. There are differences in the sanitary management in different slaughterhouses, which determine the contamination status of pathogens in the environment and final products is also different. This study selected a large broiler slaughterhouse in the Shandong Province to evaluate the contamination status of *Campylobacter* in the whole slaughterhouse process, particularly in the exposed environment, and thus to lock the key risk points for environmental sanitation control. It was found that the *Campylobacter* concentration carried in the pre-cool pond water had the greatest impact on the contamination risk of the terminal chicken products, followed by the bacterial carrying rate on the eviscerating machine. In theory, pre-cooling rinsing can reduce bacterial contamination, but unqualified hygiene can indeed make the pre-cooling pool as “a large dye tank” of bacteria, in which potential cross-contamination to each chicken is extremely serious. Obviously, the hygiene control of pre-cooling in the slaughterhouse we are studying should be specially strengthened.

The control of critical risk points is important during the slaughtering process of broilers. In May 2013, the Food Safety Administration of the United States (FSIS) issued HACCP (Hazard Analysis and Critical Control Point) system certification guidelines (17) and defined the critical risk point control measures for broiler slaughter. In September of the same year, the FSIS added *Campylobacter* control measures (18) on the basis of *Salmonella* in broilers. China also developed the corresponding HACCP in livestock and poultry slaughtering (19), but the implementation is the autonomous behavior of the slaughtering enterprise, and is currently not supervised, even some broiler slaughterhouses have not been certified and standardized for implementation. This study analyses and clarifies the critical control points of *Campylobacter* contamination in broiler slaughterhouses. The slaughtering enterprise can therefore take measures to strengthen the hygienic control of pre-cool pond water effectively, and to ensure the terminal chicken products with better quality and safety.

In conclusion, the quantitative evaluation model of *Campylobacter* contamination including environmental samples during broiler slaughtering was constructed for the first time in this study. Changes in *Campylobacter* contamination in chicken meat during slaughtering the process were identified based on the model simulation data, which highlighted that *Campylobacter* concentration carried in the pre-cool rinsing water was the critical risk control point. Finally, the model provided a theoretical basis for the sanitary control and risk management of *Campylobacter* contamination in broiler slaughterhouses.

## Data Availability Statement

All datasets generated for this study are included in the article/supplementary material.

## Ethics Statement

The animal study was reviewed and approved by Animal Ethics Committee of China Animal Health and Epidemiology Center.

## Author Contributions

GZ: model construction and manuscript drafting. XH and NL: *Campylobacter* isolation in samples. YL and LW: *Campylobacter* identification and data analysis. JZ and YG: sample and data collection from slaughter house. JuaW, ZQ, and JL: manuscript revision. JunW: research overall guidance.

## Conflict of Interest

The authors declare that the research was conducted in the absence of any commercial or financial relationships that could be construed as a potential conflict of interest.

## References

[1] Hutchison ML Taylor MJ Tchòrzewska MA Ford G Madden RH Knowles TG. Modelling-based identification of factors influencing campylobacters in chicken broiler houses and on carcasses sampled after processing and chilling. J. Appl. Microbiol. (2017) 122:1389–401. 10.1111/jam.13434 28258625

[2] Whitworth J Rise in UK Chickens Positive for top Levels of Campylobacter. Food Safety News (2019). Available online at: https://www.foodsafetynews.com/2019/06/rise-in-uk-chickens-positive-for-top-levels-of-Campylobacter/ (accessed May16, 2019).

[3] Diet and Food Every other chicken in the trade with infected diarrhoea pathogens (2019). Available online at: https://dailytophealth.com/diet-food/every-other-chicken-in-the-trade-with-infected-diarrhoea-pathogens/

[4] BIOHAZ Scientific opinion on campylobacterin broiler meat production: control options and performance objectives and/or targets at different stages of the food chain. EFSA J. (2011) 9:2105 10.2903/j.efsa.2011.2105 PMC13129689 42078736

[5] Zhao G Wang Y Wang J Research status and problem analysis of quantitative risk evaluation of pathogenic microorganisms in livestock and poultry products. Agri Product Qual Saf. (2016) 6:41–6. 10.3969/j.issn.1674-8255.2016.06.010

[6] Jarvis B Sampling for microbiological analysis. In: Lund BM, Baird-Parker TC, Gould GW, editors. The Microbiological Safety and Quality of Food, Vol. II Aspen Publishers, Inc (2000). p. 1727–28.

[7] Zhao G Liu N Zhao J Li Y Wang J Qu Z Quantitative risk evaluation of *Salmonella* contamination in broiler slaughtering and processing. China Animal Quarantine. (2018) 35:26–31. 10.3969/j.issn.1005-944X.2018.04.007

[8] Thomas NL Observations of the relationship between the surface area and weight of eviscerated carcases of chickens, ducks and turkeys. Int J Food Sci Technol. (2007) 13:81–6. 10.1111/j.1365-2621.1978.tb00780.x

[9] Gong C Wang Z Wu H Dong F Sun Y Quantitative evaluation of dietary exposure risk of Vibrio parahaemolyticus in seafood of Yantai sea area. J Food Safe Qual Inspect. (2015) 6:3485–90.

[10] Zhu H Ping Y An Y Zhou L Wang F Yan G Study on Salmonella and *Campylobacter* contamination in broiler farming and processing. Chin. Poult. (2018) 40:80–4. 10.16372/j.issn.1004-6364.2018.04.018

[11] Guo P Guo D The next challenge for the safety of food and drink Chang. pigs and poultry in animal husbandry abroad. CampylobacterVac. (2009) 29:60–1.

[12] Crotta M Georgiev M Guitian J Quantitative risk assessment of *Campylobacter* in broiler chickens - Assessing interventions to reduce the level of contamination at the end of the rearing period. Food Control. (2017) 75:29–39. 10.1016/j.foodcont.2016.12.024

[13] DanishAgricultureFoodCouncil A Quantitative Microbiological Risk Assessment of Campylobacterin the Broiler Meat Chain. EFSA Supporting Publications (2011).

[14] WHO Risk Evaluation of Campylobacterin Broiler Chickens. (2009). Available online at: https://www.who.int/foodsafety/publications/micro/MRA11_En.pdf

[15] Zhai W Jiao Y Jin L Jiao X Current situation of *Campylobacter* contamination in poultry meat and research progress of prevention and control measures. Adv Anim Med. (2010) 31:180–4. 10.3969/j.issn.1007-5038.2010.z1.046

[16] Huang J Zang X Zhai W Guan C Lei T Jiao X Campylobacterspp. in chicken-slaughtering operations: a risk assessment of human campylobacteriosis in East China. Food Control. (2018) 86:249–56. 10.1016/j.foodcont.2017.11.026

[17] FSIS FSIS. Compliance Guideline HACCP Systems Validation (2013). Available online at: https://www.fsis.usda.gov/wps/wcm/connect/a70bb780-e1ff-4a35-9a9a-3fb40c8fe584/HACCP_Systems_Validation.pdf,?MOD=AJPERES.

[18] FSIS Salmonella, and CampylobacterVerification Program for Raw Meat and Poultry Products. (2013). Available online at: https://www.fsis.usda.gov/wps/wcm/connect/ebf83112-4c3b-4650-8396-24cc8d38bf6c/10250.1.pdf?MOD=AJPERES.

[19] Chinese Ministry of Commerce GB/T 20551-2006, Application Specification for HACCP in Livestock and Poultry Slaughtering. Beijing: China Standard Press (2006).

